# Immunoregulatory Sensory Circuits in Group 3 Innate Lymphoid Cell (ILC3) Function and Tissue Homeostasis

**DOI:** 10.3389/fimmu.2020.00116

**Published:** 2020-02-06

**Authors:** Rita G. Domingues, Matthew R. Hepworth

**Affiliations:** Division of Infection, Immunity and Respiratory Medicine, Faculty of Biology, Medicine and Health, Manchester Collaborative Centre for Inflammation Research, Lydia Becker Institute of Immunology and Inflammation, Manchester Academic Health Science Centre, The University of Manchester, Manchester, United Kingdom

**Keywords:** innate lymphoid cells, ILC, mucosal immunology, neuroimmune, circadian, immune circuits

## Abstract

Recent years have seen a revolution in our understanding of how cells of the immune system are modulated and regulated not only via complex interactions with other immune cells, but also through a range of potent *inputs* derived from diverse and varied biological systems. Within complex tissue environments, such as the gastrointestinal tract and lung, these systems act to orchestrate and temporally align immune responses, regulate cellular function, and ensure tissue homeostasis and protective immunity. Group 3 Innate Lymphoid Cells (ILC3s) are key sentinels of barrier tissue homeostasis and critical regulators of host-commensal mutualism—and respond rapidly to damage, inflammation and infection to restore tissue health. Recent findings place ILC3s as strategic integrators of environmental signals. As a consequence, ILC3s are ideally positioned to detect perturbations in cues derived from the environment—such as the diet and microbiota—as well as signals produced by the host nervous, endocrine and circadian systems. Together these cues act in concert to induce ILC3 effector function, and form critical sensory circuits that continually function to reinforce tissue homeostasis. In this review we will take a holistic, organismal view of ILC3 biology and explore the tissue sensory circuits that regulate ILC3 function and align ILC3 responses with changes within the intestinal environment.

## Group 3 Innate Lymphoid Cells—Sentinels of the Gastrointestinal Tract

Innate lymphoid Cells (ILCs) are a family of innate immune effectors that localize mainly to mucosal surfaces and which play critical roles in regulating tissue immunity and homeostasis. The ILC family can be divided into three main subsets—group 1 ILC (ILC1), ILC2, and ILC3 based on their expression of master transcription factors and associated effector cytokine profiles [Reviewed extensively elsewhere (1–8)]. In this review we will focus on group 3 ILC (ILC3), a group of ILC that act constitutively to maintain intestinal health through regulation of the intestinal barrier and commensal microbiota, and through protective immune responses against extracellular microbial pathogens.

ILC3s are characterized by the expression of the retinoid-related orphan receptor γt (RORγt) (1, 5, 6) and they can be further sub-divided into at least two sub-groups in adults (9). These subsets are developmentally, transcriptionally and functionally heterogeneous and include lymphoid tissue inducer cells (LTi)-like ILC3s; characterized by surface expression of CCR6, c-kit (CD117), Neuropilin-1, and variable expression of CD4, in addition to natural cytotoxicity receptor expressing (NCR)+ ILC3s—which lack LTi-associated markers but express a range of NCR (e.g., NKp46 in mice) while further co-expressing the transcription factor T-bet (10, 11). The characteristics and differences between ILC3 subsets have been discussed in detail elsewhere (9) and as such, for the sake of clarity, we will largely refer to ILC3 cumulatively in this review without distinguishing the specific subset.

**Figure 1:**
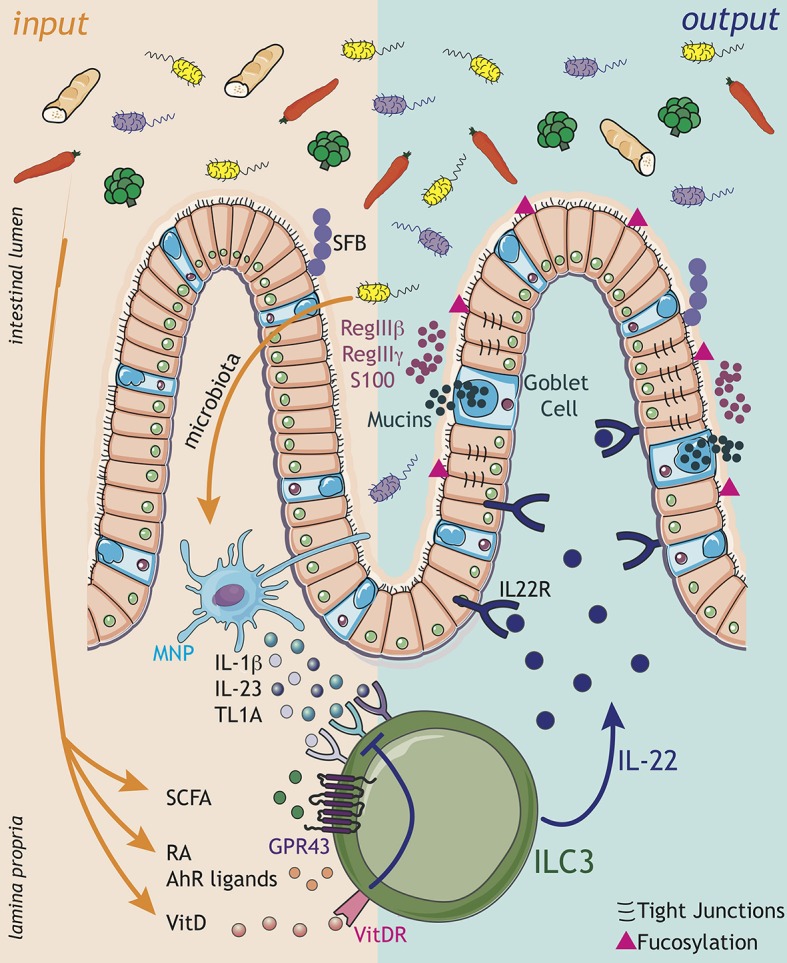
ILC3 engage in complex sensory circuits in order to integrate microbial and dietary cues and enforce mucosal homeostasis. **Inputs** (orange arrows): ILC3s act as innate immune sentinels of the gastrointestinal tract, and respond rapidly to changes in the tissue environment. Environmental signals, comprising microbial and dietary cues, are sensed either via myeloid cell intermediaries [e.g., dendritic cells (DC), macrophages, also known as mononuclear phagocytes (MNP)], which release cytokine cues (IL-1β, IL-23, TL1A) to modulate ILC3 function, or through direct sensing of metabolites and dietary ligands. Microbial metabolites, such as short chain fatty acids (SCFA), signal directly to modulate ILC3 function though the receptor GPR43. Additionally, ILC3 integrate dietary cues in the form of the vitamin A metabolite retinoic acid (RA) and AhR ligands, which together promote ILC3 development and effector cytokine responses. In contrast, vitamin D acts as a negative regulator of ILC3 activation by suppressing the ability of ILC3 to sense myeloid cues—such as IL-23. Within the complex tissue microenvironment ILC3 are likely exposed to multiple signals in parallel, which must be appropriately integrated to maintain intestinal homeostasis. **Outputs** (dark blue arrows): Signals translated by ILC3 are propagated in the form of ILC3-derived *outputs*—most notably cytokine signals, which are received by other immune and non-immune cells within the local environment. In particular, ILC3-derived IL-22 acts on epithelial cells to enforce intestinal barrier integrity and induce the production of antimicrobial peptides (AMPs) such as RegIIIβ, RegIIIγ, and S100 family proteins, secretion of mucins by goblet cells, modulation of tight junctions and epithelial cell fucosylation. IL-22-dependent pathways further regulate the growth of specific commensal bacteria species that are intimately associated with the host, such as segmented filamentous bacteria (SFB). Together, the balance of signals perceived by ILC3 determine the strength of the effector response, regulate the balance of the commensal microbiota and ensure their spatial segregation from the underlying intestinal tissue. In the context of disease, dysregulation of these signals may dramatically alter ILC3 responses and result in a loss of barrier function and translocation of the bacteria from the lumen, thus precipating or exacerbating inflammatory disease.

As discussed in detail below, ILC3s are at the center of multiple tissue regulatory circuits in which a variety of *inputs* (in the form of environmental and host-derived cues) are sensed and interpreted by ILC3 and give rise to functional *outputs* that culminate in the downstream modulation of tissue physiology to maintain health and homeostasis. While the *inputs* of these sensory circuits vary, and will be discussed in detail below, a major common ILC3-associated *output* is the secretion of effector cytokines including IL-22, IL-17A, IL-17F, and GM-CSF and lymphotoxin (LT) (1, 4, 7, 8) (Figure 1). These soluble mediators in turn act upon both neighboring tissue-resident immune cells and non-hematopoietic cells—such as epithelia and stroma. In this review, we will comprehensively discuss the major tissue circuits through which ILC3 function is regulated, and through which ILC3 propagate these signals to regulate and orchestrate the wider immune response and to promote optimal tissue function, mediate protective immune responses and maintain health.

## ILC3 Circuits in the Regulation of Intestinal Homeostasis

### Host-Microbiota Sensory Circuits

Mammals have evolved multiple complimentary immunological mechanisms to promote the anatomical containment of commensal bacteria. These mechanisms enforce tolerance, suppress inflammation and maximize mutualism with the microbiota, and ILC3s have key roles in this process (12–15). ILC3s are enriched within gastrointestinal (GI) tract where they are ideally positioned to promote barrier repair and to prevent bacterial translocation (15). ILC3 produce a range of soluble mediators that enable them to continually reinforce the barrier and maintain the containment and physical segregation of commensal microorganisms. Chief amongst these mediators is the cytokine interleukin (IL)-22, which binds to the heterodimeric receptor IL22RA1-IL10RB (IL-22R) expressed by cells of the non-haematopoietic lineage, most notably intestinal epithelial cells (Figure 1: *outputs*). IL-22 signaling induces the production of antibacterial peptides such as RegIIIβ and RegIIIγ and S100 family members, which in turn regulate the commensal microbiota and limit access to the epithelial and mucosal niche (16, 17). IL-22 also promotes the physical exclusion of commensal bacteria through induction of mucins and goblet cell hyperplasia, and by regulating the expression of tight-junction components (15, 17, 18). Moreover, ILC3s induce fucosylation of intestinal epithelial cells through an IL-22 and LTα driven process, which in turn favors colonization by mutualistic bacterial species at the expense of potential pathogens (Figure 1: *outputs*) (19–21). In addition, IL-22 produced by ILC3s acts to regulate epithelial turnover and intestinal crypt stem cell maintenance, and has been ascribed both pro- and anti-tumorogenic functions, most recently being shown to promote DNA damage response (DDR) mechanisms in order to prevent tumor formation (22–25). IL-22 also modulates nutrient uptake via the intestinal epithelia, in particular lipid uptake (26). In line with this central role for ILC3 and IL-22 in maintaining intestinal barrier function and tissue homeostasis, loss of IL-22 production by ILC3s in mice results in dysbiosis, barrier disruption and an increased susceptibility to experimental induced colitis (27, 28). Moreover, depletion of intestinal ILC3 leads to peripheral dissemination of intestinal bacteria and systemic inflammation that can be rescued by providing exogenous IL-22 (15). Thus, a central function, and key *output*, of ILC3-mediated effector responses is the orchestration of host-microbiota interactions (Figure 1: *outputs*).

Intestinal homeostasis and host-commensal interactions are also modulated by the type 3 cytokines IL-17A and IL-17F, both of which are also produced by ILC3 (1, 4, 7, 8). Similar to IL-22, IL-17A/F promote tissue integrity by enhancing the synthesis of tight junctions and antimicrobial peptides, including β-defensins, REG proteins, S100 proteins, lipocalins and lactoferrins (29). Additionally IL-17A/F act in part to attract myeloid cells to the tissue site, through the induction of chemokines and growth factor expression by epithelial cells (30, 31). While ILC3 have been reported to be a potent source of IL-17A/F in early life, expression of these cytokines appears to be somewhat limited at steady state in adult tissues (28, 32). In contrast, during infection and inflammation ILC3 produce IL-17 in response to myeloid-derived cues including IL-23 and IL-1β (33, 34), and ILC3-derived IL-17 has been attributed critical roles in immunity to fungal and bacterial pathogens (34–37). In particular, IL-17 production by ILC3s has been implicated in immunity against fungal pathogens, specifically in response to *Candida albicans* (34). Interestingly, HIV patients commonly manifest oropharyngeal candidiasis, and loss of IL-17 production by ILC3s was observed in tonsils and buccal mucosa during SIV infection in macaques (38, 39).

While homeostatic IL-17 production has been attributed protective functions in intestinal health and host-commensal microbe interactions, elevated IL-17A/F production has also been associated with the pathogenesis of inflammatory bowel disease (IBD). Indeed, ILC3-derived IL-17A and IL-17F are increased during intestinal inflammation in both mice and humans (40, 41). Together, IL-17A/F production by intestinal ILC3—in addition to Th17 and γδ T cell populations—has highly contextual roles in intestinal health, immunity and inflammation.

Conversely, the microbiota itself is also increasingly appreciated to act reciprocally to modulate ILC3 function (Figure 1: *inputs*). Indeed, early studies suggested microbial colonization of the neonatal intestine regulates the composition and size of the ILC3 pool within the intestinal tract. Pups born to germ free mothers were reported to have reduced frequencies of ILC3s—indicating a role of microbial signals in promoting tissue seeding by ILC3 subsets (28). However, in contrast to these findings IL-22 producing ILC3 numbers were found to be suppressed in a microbiota dependent manner through epithelial expression of IL-25 (32). Despite these discrepancies, the dialogue between the microbiota and ILC3s within the intestine has emerged as a critical circuit of intestinal immunity and tissue homeostasis.

Recent studies have begun to shed light on the microbial-derived metabolites that mediate this immune regulatory on ILC3. For example, ILC3s have the capacity to sense and respond to short chain fatty acids (SCFA)—including butyrate, acetate and propionate—critical regulators of immune responses which are metabolized from dietary fiber by commensal microbes (Figure 1: *inputs*) (42, 43). Levels of butyrate differ along the intestinal tract, in line with differing densities of commensal microbes, and were previously correlated with reduced ILC3 cell number and cytokine production in distal regions of the small intestine (44). SCFA can signal via multiple G-coupled protein receptors, as well as via histone deacetylase enzymes (HDAC) (43), and despite these advances the mechanisms through which SCFA regulate ILC3 are still being delineated. The SCFA receptor GPR109a was implicated in the microbiota-associated regulation of ILC3 cytokine production via the modulation of dendritic cell (DC)-derived IL-23 in the colon, although these studies largely utilized a GPR109a agonist—leaving the precise contribution of endogenous SCFA unclear (45). Interestingly, a recent study highlighted ILC3-intrinsic expression of the SCFA receptor *Gpr43* (*Ffar2*) in the modulation of intestinal ILC3 responses (Figure 1: *inputs*) (46). Triggering of GPR43 with the SCFAs propionate and acetate (but not butyrate) selectively promoted colonic ILC3 proliferation and expansion and production of IL-22, subsequently protecting mice from chemically induced colitis and from enteric bacterial infection (46).

### Dietary Circuits

Cues derived from mutualistic microbiota establish a critical dialogue between the host and it's environment and regulate the intestinal immune system—including ILC3. In addition to the microbiota, the intestine is also continually exposed to metabolites and phytochemicals derived from the diet (Figure 1: *inputs*). As highlighted above, the availability and liberation of many dietary metabolites is also determined in part by mutualistic, commensal microbes within the intestine—while conversely the diet itself can modulate microbial composition and thus, determine the nature of host-commensal interactions. For example, the feeding of high fat diet (HFD) to pregnant mice was found to modify the expansion of ILC3 in the intestines of progeny through the modification of the mothers microbiota (47).

Similarly, Aryl hydrocarbon receptor (AhR) ligands are normally liberated from cruciferous vegetables in the diet—such as broccoli and cabbage, but they can also be microbially derived (48). AhR is a dietary-sensing nuclear receptor that is expressed by ILC3s and has critical roles in the development, transcription and function of these cells (Figure 1: *inputs*). Indeed, ILC3 are highly AhR dependent, and present severe functional impairments in the absence of cell-intrinsic AhR expression (14, 49–51). As a result of ILC3 defects, AhR-deficient mice fail to form tissue-associated lymphoid structures, such as cryptopatches (CP) and are unable to control infections with the extracellular pathogen *Citrobacter rodentium* (49, 52). Intriguingly, the development and seeding of intestinal ILC3 in neonates was demonstrated to be dependent upon the mothers microbiota and the transfer of antibody-bound AhR ligands through the mothers milk (48), suggesting maternal transfer of dietary ligands to neonates may play critical roles in the development of the immune system, microbial colonization and protection from infections in early life.

Indeed, maternal transfer of dietary ligands is increasingly appreciated to be a determinant of neonatal immunity and ILC3 development. *In utero* exposure to the Vitamin A metabolite retinoic acid (RA) impacts directly on secondary lymphoid organ development with long-term immunological consequences (53). Mice genetically modified to have hematopoietic cell-intrinsic deficiency in RA lacked PP or exhibited impairment in LN formation and maturation as a result of defective ILC3 differentiation (Figure 1: *inputs*). Moreover, it was shown that RA directly regulates the master transcription factor of ILC3, RORγt, and in the absence of maternal retinoids ILC3 failed to develop correctly (53). In addition to maternally derived RA signals, deprivation of vitamin A in adulthood also results in the collapse of the intestinal ILC3 populations and, as a consequence, results in susceptibility to *Citrobacter rodentium* infection (54, 55). In addition to direct effects of RA on ILC3 development, RA produced by DCs was also found to regulate the homing properties of ILC3s by imprinting expression of the intestinal homing markers CCR9 and α4β7 (56).

The importance of dietary vitamins in ILC3 effector circuits is further supported by evidence that vitamin D also plays a role in intestinal ILC3 homeostasis (Figure 1: *inputs*). ILC3 numbers in the small intestine of mice deficient for the vitamin D receptor (VDR—KO mice) were shown to be increased, as was IL-22 expression, resulting in enhanced resistance to infection with *Citrobacter rodentium* (57). Consistently, human ILC3s stimulated with IL-23 and IL-1β upregulate the VDR, and VDR signaling subsequently acts to downregulate the IL-23 signaling pathway—suggesting vitamin D acts as a negative regulator and suppressive feedback loop to control ILC3 activation (Figure 1: *inputs*) (58). Vitamin D availability has also been implicated in the pathogenesis of IBD, as patients are reported to have lower plasma levels of vitamin D than healthy subjects, and exhibit an upregulation of the IL-23 signaling pathway which could potentially explain exacerbated ILC3 responses that are associated with intestinal inflammation in IBD (58). In contrast to these studies, mice lacking *Cyp27B1—*an enzyme required for the conversion of vitamin D to it's chemically active form—exhibit reduced colonic ILC3 numbers and IL-22 production suggesting a more nuanced role for vitamin D in the regulation of ILC3 function (59). Together these findings highlight the importance of dietary cues in regulating ILC3 function and intestinal homeostasis. An increased understanding of the complex dialogue between diet, microbiota and host is likely to reveal novel immune regulatory circuits and clarify how environmental cues act as risk factors, and contribute to the onset of metabolic and inflammatory disorders.

## ILC3 Immune Crosstalk in the Orchestration of Intestinal Health

### Translating Microbial Cues: Myeloid—ILC3 Circuits

While ILC3 are potently regulated by the microbiota and diet within the intestinal environment, it remains unclear the extent to which they are able to directly sense these cues, beyond the pathways detailed above. Indeed, the majority of evidence suggests third party sensory cells of the myeloid lineage are required to directly sense, translate and communicate environmental information to ILC3. Classically, tissue-resident mononuclear phagocytes (MNPs) act as key intermediaries and signal to ILC3 via the release of cytokine mediators during both homeostatic and protective immune responses (60, 61). Indeed, intestinal myeloid populations are well-equipped to directly sense microbial metabolites, pathogen associated molecular patterns (PAMPs) and danger signals and to transfer this information to ILC3 (Figure 1: *inputs*). In particular, CX3CR1^+^ intestinal MNPs cluster with ILC3 in distinct, organized lymphoid structures, such as CPs (62, 63). Microbiota sensing by CX3CR1^+^ MNPs was shown to result in local production of IL-1β and IL-23, which are key activating cytokines of ILC3 and which potently induce IL-22 secretion (Figure 1: *inputs*) (63). Depletion of CX3CR1^+^ MNPs resulted in impaired IL-22 production by ILC3 and failure to control *Citrobacter rodentium* infection (62, 64, 65). In addition to the provision of the activating signals IL-23 and IL-1β, CX3CR1^+^ MNP-derived TL1A further acts to augment IL-22 production from ILC3 (Figure 1: *inputs*) (62).

## Neuroimmune Circuits

While microbial sensing by intestinal MNP and conserved crosstalk with ILC3 appear to be a major sensory circuit of intestinal immunity, emerging evidence suggests diverse sensory mechanisms across multiple biological systems provide additional *inputs* to regulate ILC3 function. In particular, the central and enteric nervous systems are rapidly being appreciated as critical sensory and immunoregulatory systems.

It has been suggested that the immune and nervous systems are evolutionary linked, since they share functional similarities (66, 67). Both nervous and immune systems rely on similar processes to for cellular communication; such as cell-cell contact and synapse formation, release of soluble mediators and sensing of circulating metabolites. Recent evidence suggests immune and neuronal cells are positioned in close proximity, and form conserved interactions that have been termed “neuro-immune cell units” (NICUs) (67). NICUS can form through interactions with both the central and peripheral nervous system and are increasingly being described in peripheral tissues such as the gastrointestinal tract and lung.

**Figure 2:**
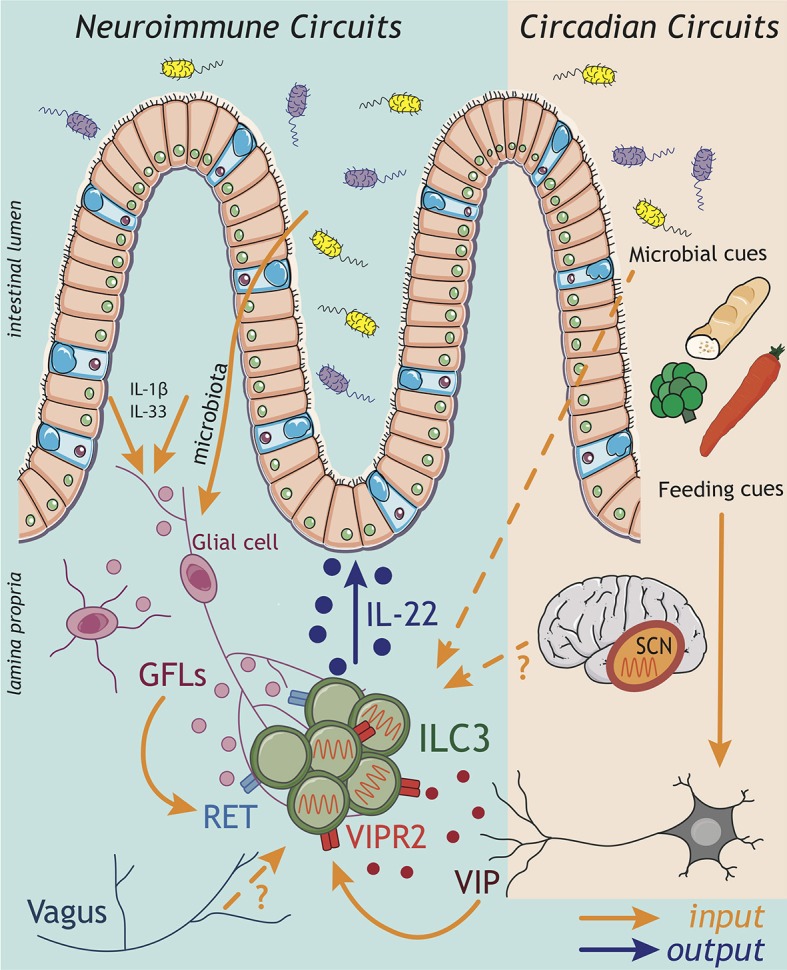
ILC3 neuroimmune and circadian circuits. Emerging findings implicate *inputs* from the nervous system in the regulation of ILC3 circuits within the gastrointestinal tract. Both the central (CNS) and enteric (ENS) nervous systems have the capacity to sense perturbations within the intestinal environment and relay this information via the release of neuropeptides to influence the ILC3 response. Strikingly, enteric glial cells are able to directly sense microbial patterns and alarmins released within the tissue, and respond by producing glial-derived neurotrophic factors (GDNF family of ligands; GFL) that directly activate the production of IL-22 by ILC3 through the tyrosine kinase RET. Indeed, recent evidence suggests a broader spectrum of neuropeptides may act to regulate ILC3 function including vasointestinal peptide (VIP) produced by enteric neurons in response to feeding cues. Signals transmitted by the nervous system also play critical roles in aligning ILC3 effector function with periods of activity and high risks of environmental exposure and pathogen encounter over the course of a 24 h day. In this regard, circadian rhythms entrained by light—and sensed via the suprachiasmatic nucleus (SCN) of the brain—trigger a cascade of molecular transcriptional-translational feedback loops of clock genes, which orchestrate rhythms in the ILC3 response. While the “central clock” within the CNS appears to be a central entrainer of ILC3 oscillatory function in the gut, the mechanisms through which the CNS transmits this information to regulate ILC3 function peripherally remain unknown. Nonetheless, *inputs* from the CNS have previously been shown to be relayed to ILC3 via the vagus nerve. Together cues from both the CNS and ENS have the potential to entrain intestinal ILC3 function, while circadian rhythms in ILC3 may be imprinted through a combination of central clock-mediated light entrainment, feeding-associated neuronal feedback and environmental cues from the microbiota.

Neuroimmune interactions are evident very early in life—and during the embryonic period the development of the enteric nervous system (ENS) and SLO organogenesis share many parallels. Notably, the neurotrophic factor receptor RET is essential for the development of Peyer's patches (PP) and also the ENS (68, 69). Moreover, RET expression by CD11c^+^ cells present in the anlagen initiates a cascade of immune cell recruitment, in particular of fetal ILC3s, through sensing of neurotrophic factors that drive the formation of primordial lymphoid clusters (68, 69). Moreover, increasing evidence suggests ILC3 can directly sense these neuronal derived *inputs* and respond during both development as well as in the adult intestine (Figure 2: *inputs*). As mentioned previously, fetal and adult ILC3 development and function relies on RA signaling (53). Intriguingly, neurons have been suggested to be a physiological source of RA (70), surprisingly suggesting RA may be derived not only from the diet but also from the host nervous system.

The ENS is increasingly appreciated to regulate tissue-resident immune functions (71), include those of ILC3. One pioneer study demonstrated that a glial-ILC3-epithelial axis is required to regulate enteric defense against bacterial infection (72). Like myeloid cells, intestinal glial cells also have the capacity to sense microbial cues and alarmins in a *Myd88*-dependent manner; thus, implicating the enteric nervous system as a key player in environmental sensing circuits. In response to these cues glial cells secrete neurotrophic factors, which directly act on adult ILC3 cytokine production via cell-intrinsic RET expression (Figure 2: *inputs*). Ablation of *Ret* in ILC3s led to a reduction in IL-22, consequently impairing epithelial function and host defense to enteric bacterial infection (72). In addition to ENS cues, CNS–derived signals propagated by the vagus nerve—via release of acetylcholine—have also been implicated in the regulation of ILC3 responses to bacterial infections in the peritoneal cavity (73). Vagal disruption was shown to lead to dysregulated ILC3 cell numbers in the peritoneal lavage (73). Mechanistically, acetylcholine acted to promote the release of pro-resolving lipid mediators—generated via ILC3-intrinsic expression of the PCTR biosynthetic pathway—which subsequently promoted protective immunity during *E. coli*-driven sepsis (73). Together, these studies illustrate the importance of neuronal *inputs* in regulating ILC3 *outputs* during infection (Figure 2).

Recent studies suggest that the number of neuropeptides with immunoregulatory activity may be broader than previously appreciated. Vasoactive intestinal peptide (VIP) release by enteric neurons was also shown to regulate ILC3-derived IL-22 production through signaling via VIPR2, triggered in part by feeding and dietary cues (Figure 2: *inputs*) (74, 75) (discussed in detail below). However, despite the strategic location of ILC3s within the CPs, which are enveloped by glial cell nervous fiber bundles and neuronal projections, the full extent of neuroimmune interactions that regulate ILC3 function are still to be determined. Indeed, recent years have seen an explosion in our understanding of neuroimmune signals that regulate other immune cells, including other members of the ILC family—most notably ILC2s (76–82). These studies have opened up new avenues of research and expanded our understanding of crosstalk between diverse biological systems, thus provoking the need for further studies to fully elucidate neuroimmune sensory circuits in the regulation of ILC3 responses, intestinal immunity and host-microbiota interactions.

## Anticipatory ILC3 Responses and Circadian Circuits

In addition to local environmental cues, mammals are also constantly exposed to a range of external stimuli and pressures such as fluctuations in temperature, oxygen levels and the daily light cycle. As a result many organisms have evolved circadian rhythms to align core biological processes with time of day, which are imprinted by an internal biological clock. Specifically, circadian rhythms are driven by cell-autonomous transcriptional feedback loops (“clocks”), which enable organisms to anticipate and adapt to temporal changes in their environment (e.g., changing seasons, jet lag, shift work) and regulate metabolically demanding biological processes including body temperature, locomotor activity, endocrine responses, and feeding behavior—while on the cellular level circadian clocks regulate cellular metabolism and cell cycle (83, 84). In line with this, it is increasingly appreciated that circadian rhythms also regulate immune cell responses (85), and immune cells exhibit circadian oscillations in leukocyte trafficking, priming, effector function and host-pathogen interactions (85).

In mammals, circadian rhythms are controlled by the central circadian pacemaker or master clock—located in the suprachiasmatic nucleus (SCN) of the brain (86). The SCN acts to interpret and propagate light cues received via the optical nerve and subsequently, cell autonomous circadian rhythms are imprinted by systemic signals that act to align oscillations in a tripartite system of transcriptional-translational feedback-loops (85, 87, 88). The induction of the loop starts with the transcriptional activators CLOCK and BMAL1 promoting the expression of the repressors Period (*Per*) and Cryptochrome (*Cry*), which in time translocate back into the nucleus and inhibit their own expression (85, 87, 88). The second loop is composed by nuclear receptors RAR-related orphan receptors (RORs) (α, β, γ) and REV-ERBs (α, β), which exert opposing effects on the clock through transcription factor binding to the promoter of *Arntl* (encoding BMAL1) (85, 87, 88). Finally, the third loop consists of transcriptional activator albumin D-box binding protein (DBP) and the repressor nuclear factor for interleukin 3 (NFIL3), which act synergistically to regulate the expression of D-box genes including that of *Per* (85, 87, 88). Upon establishment of the transcriptional loops, the SCN keeps peripheral clocks in synchrony via neuronal sympathetic/parasympathetic transmission and through the hypothalamus pituitary adrenal (HPA) axis, including the release of catecholamines (epinephrine and norepinephrine) and glucocorticoids (84). Remarkably, similar circadian molecular mechanisms are found in the periphery. However, while the SCN network allows for the generation of sustained oscillations and time-of-day alignments, perturbations from environmental *inputs* such as temperature changes, the microbiota and feeding cues can also impact on peripheral, cell-intrinsic clocks (84).

Many constitutive innate immune processes, including the maintenance of intestinal barrier function via steady state IL-22 release from ILC3, come with significant metabolic costs for the host. Thus, circadian rhythms are thought to have evolved to align these processes with anticipated challenges and times of highest risk—most notably during waking activity and feeding where exposure to microbes, dietary antigens and potential pathogens is highest. Intriguingly, several components of the transcriptional circadian clock machinery including NFIL3 and RORγ/α are also key transcriptional regulators of ILC3 development and function, suggesting the possibility that these cells may also be regulated in a circadian manner (89–92). Moreover, ILC3 and IL-22 are critical regulators of the intestinal microbiota, with oscillations also reported amongst levels of commensal microbes in the intestinal tract (93, 94).

In line with this, several recent studies have demonstrated circadian control and oscillatory ILC3 responses, which are regulated by the master clock gene *Arntl* (Bmal1) in a cell-autonomous fashion (95, 96). Deletion of *Arntl* in ILC3s resulted in an altered epigenetic landscape, dysregulated cell numbers and IL-22 expression, and subsequently contributed to alterations in steady-state oscillations in the microbiome itself (95–97). Moreover, disrupted ILC3 responses resulted in altered epithelial responses and disrupted lipid uptake within the intestine (74, 95, 96). Of note, while deletion of *Arntl* led to a broad impairment of total ILC3 numbers (95), deletion of the related clock gene *Nr1d1* (also known as *Rev-erb*α) resulted in altered ILC3 subset development—with mice exhibiting a marked reduction NCR^+^ ILC3s, while LTi-like ILC3 were unperturbed (97). Moreover, lack of *Nr1d1* increased expression of *Il17* in ILC3s, a mechanism previously reported in in Th17 cells (97, 98). Interestingly, ILC3s isolated from the inflamed intestine of patients with IBD presented with alterations in expression of several circadian-related genes, including *Nr1d1*, suggesting circadian clock disruptions—such as those seen in shift workers—may act to disrupt normal immune function and have relevance in the onset and/or pathogenesis of chronic inflammatory diseases (96). Of note however, the role of *Nr1d1* as a transcriptional regulator of both *Nfil3* and *Rorc* suggests clock-associated genes may have additional roles that are independent of circadian regulation (97).

Circadian rhythms may be imprinted by a range of systemic and environmental cues. Within the intestinal tract feeding cues were shown to contribute to the entrainment of oscillatory function in ILC3 (Figure 2: *inputs*). Time-specific feeding altered daily circadian rhythms in clock related genes (95) and IL-22 expression oscillated across the day between active and resting phases (74, 75). Interestingly, signals from the ENS appear to be critical in sensing feeding cues to entrain circadian rhythms in ILC3 (Figure 2) (74, 75). Feeding was found to induce VIP release from enteric neurons, consequently triggering VIPR2 signaling in ILC3s and *enhancing* IL-22 production and the barrier function of the epithelium. In contrast during fasting this neuropeptide cue waned, resulting in decreased IL-22 production by ILC3s and thus, imprinting diurnal rhythms onto intestinal ILC3s (74). In contrast, another study (pre-print currently under review) reported that VIP release from enteric neurons upon feeding rather *decreases* production of IL-22 by ILC3s, allowing for the outgrowth of the epithelial-associated commensal microbe SFB (75). Despite these discrepancies, both studies clearly implicate the sensing of feeding cues by the enteric nervous system as a key entrainer of circadian rhythmicity in ILC3. One possible explanation for the apparent differences in these findings is that complex interplay with the host microbiota may further augment ENS cues or act directly on ILC3 to provide complimentary or competing *inputs*, which then combine with cues from the central clock to tune anticipatory rhythms. In line with this, the microbiota was also shown to have an impact on circadian gene expression in ILC3s—adding another layer of complexity in the crosstalk between ILC3s and the commensal microbiota (95, 96).

While these studies all implicate peripheral cues in the entrainment of anticipatory ILC3 responses, light signals derived from the central clock (in the brain) are also known to be central in aligning many biological processes and in imprinting circadian rhythms. Indeed, signals from the central clock were shown to be a key regulator of ILC3 rhythmicity (Figure 2: *inputs*) (95). Utilizing mice in which the central clock was surgically ablated, or mice genetically deficient for *Arntl* only in the SCN, ILC3s developed disrupted cytokine oscillations and an altered phenotype—including the downregulation of intestinal homing markers which could partially explain time of day differences in ILC3 numbers within the gastrointestinal tract (95). The mechanisms through which the central clock in the SCN mechanistically aligns biological processes with light cues vary, but can include the release of hormonal cues—most notably glucocorticoids (84). While it remains to be determined whether this mechanism acts on ILC3 in the context of the central circadian clock, glucocorticoids have been shown to suppress ILC3s IL-22 production *in vitro* (99). Together these findings suggest that long-range and local circadian cues may directly regulate ILC3 numbers and function during homeostasis or following infection, mediating ILC3 interactions with the microbiota and regulation of intestinal barrier function.

## Lymphoid Organogenesis: ILC3-Stromal Circuits

Unlike cells of the adaptive immune system, ILC3 are one of the first immune cells to colonize the intestine during the embryonic period and are critical for the formation of SLOs (100). In this regard one of the most fundamental circuits through which ILC3 contribute to barrier immunity is through the orchestration of organized interactions between the innate and adaptive immune system. In contrast to the sensory circuits described above, where *inputs* derived from third party cells stimulate *outputs* in ILC3, during both embryogenesis and adult life ILC3 provide the *input* and stimulatory cues to stromal cells to initiate a cascade of events that lead to the formation of secondary and tertiary lymphoid tissues.

The formation of LN and PP is initiated via specialized stromal cells, known as lymphoid tissue organizer cells (LTo) that start to express chemokines such as CXCL13, CCL19, CCL21, as well as the adhesion molecules VCAM-1, ICAM-1, and MadCAM-1 (101, 102). The expression of these factors creates a gradient to recruit *bona fide* fetal lymphoid tissue inducer cells (LTis; fetal members of the ILC family, referred to here as fetal ILC3), which cluster with the LTo forming the primitive anlagen of the SLO (103). Fetal ILC3s at this stage express CXCR5, CCR7, and α4β7; homing markers that are important for fetal ILC3 recruitment and which were shown to mediate migration toward LTo-derived chemokines and adhesion molecules, respectively. In fact, full maturation of LTo and development of lymphoid tissue is dependent on recruitment of fetal ILC3s and provision of lymphotoxin (LT) (103, 104). Conversely, LTo also provide critical survival signals for fetal LTi/ILC3 with IL-7 expression shown to be necessary for ILC3 maintenance, while IL-7R blockade in adults also resulted in a rapid loss of normal migration of B and T cells to the LN (105). This stromal IL-7 circuit is likely also active at other sites such as in the fetal liver and bone marrow, where stroma derived IL-7 signaling could trigger the expression of NFIL3 (91). In addition, the same stromal-ILC3 circuit acts to restore normal lymph node architecture following infection-induced disruption of lymphoid microanatomy (106). Therefore, the crosstalk between ILC3s and lymph node-associated stroma is reactivated in in adulthood and crucial to enable adaptive immune responses during secondary infections (106). Thus, a key sensory circuit and stimulatory loop formed between ILC3 and stromal cells is critical for the formation of lymphoid tissues, and to facilitate the action of the broader innate and adaptive immune system.

Postnatally, a large number of organized lymphoid structures designated as tertiary lymphoid structures start to form under the influence of environmental stimuli. These immune cell clusters include cryptopatches (CP), which are confined to bottom of the crypts within the intestinal lamina propria. CP formation is driven through similar molecular mechanisms to SLO, including via interactions between ILC3-associated LTα1β2 with the LTβR expressed by stromal cells and IL-7 signaling (107, 108). CPs can further give rise to isolated lymphoid follicles (ILFs) in a CCR6 and LTα1β2-dependent manner (109, 110), resulting in up-regulation of secretory antibody (Immunoglobulin A; IgA) synthesis in response to changes in the composition of microbiota (111, 112).

A unique feature of ILF development in comparison to LN, PP, and CPs is the requirement for microbial exposure. Intestinal bacteria are sensed by myeloid cells which increase the interactions between ILC3s and LTos, also via a LTα1β2 dependent axis, leading to increased expression of adhesion molecules by the stroma and recruitment of B cells to these structures (113, 114). ILFs are largely absent in a microbiota free environment, and are restored upon recolonization with commensal microbes (115). Similarly inflammation and intestinal barrier disruption results in increased numbers of ILFs in the colon, and intriguingly mice deficient in the transcription factor RORγt develop more ILFs than their wild type counterparts in the context of intestinal inflammation, suggesting a potential regulatory role for type 3 immune responses, such as ILC3, in this setting (116).

Interactions between ILC3 and stroma also provide important cues to localize ILC3 to defined tissue microenvironments, and to facilitate interactions with adaptive immunity (discussed in detail below). Within the intestine-draining mesenteric lymph node multiple distinct stromal populations have been identified with differential capacities to attract immune populations and orchestrate immune cell crosstalk (117). One such population expresses the enzyme *Ch25h*, which acts to generate the cholesterol metabolite 7,α25-OH—a key migratory ligand for multiple immune cells including ILC3 (117–120). This stromal cue is sensed by ILC3 via the receptor EBI2 (*Gpr183*), and facilitates localization of not only ILC3 but also T follicular helper cells, DCs and B cells to the follicular border of lymph nodes (118, 120–125). Similarly, within the intestinal tissue stromal generation and breakdown of cholesterol ligand cues create a migratory gradient required to recruit ILC3 to CP in a *Gpr183*-dependent manner (119). Together these studies indicate that a stromal ILC3 circuit is a key regulator not only of lymphoid organogenesis but also of ILC3 localization and function, which together facilitate the interactions between ILC3 and adaptive immune cells and foster modulatory crosstalk.

## Circuits of Immune Orchestration: Crosstalk Between ILC3 and Adaptive Immunity

ILC3s are also emerging as key orchestrators and regulators of adaptive immune responses [Reviewed in detail in (126)]. This regulation is mediated by ILC3 either through indirect modulation of bystander cells that subsequently modulate the adaptive immune response or directly via both soluble mediators and cell contact-dependent interactions with adaptive lymphocytes.

**Figure 3:**
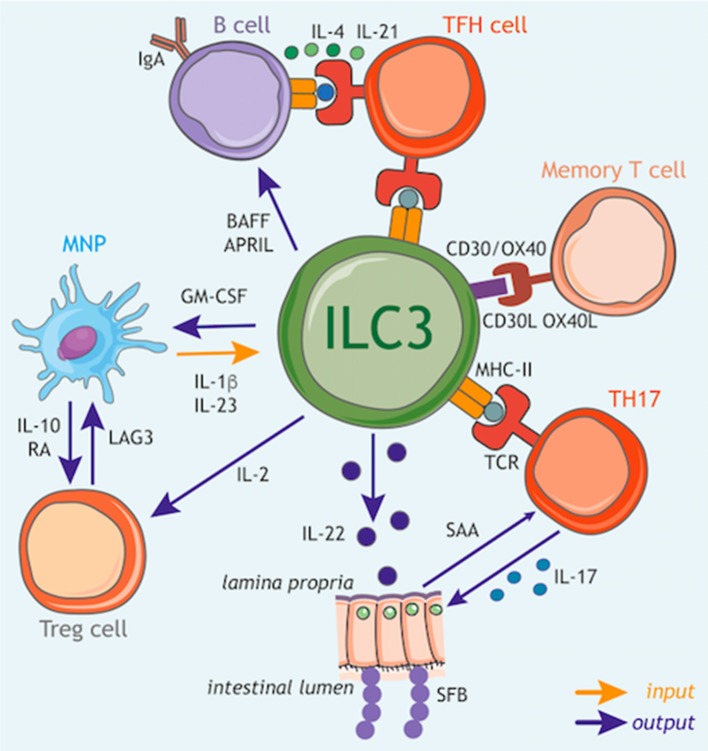
ILC3 circuits orchestrate adaptive immune responses. In addition to their function as tissue-resident cytokine producing cells, ILC3s have the capacity to participate in multiple cellular circuits through direct cell–cell modulation of T cell responses, as well as the release of soluble mediators that augment adaptive immune function and development. ILC3s can control the magnitude and quality of the CD4^+^ T cell response via antigen presentation in the context of MHC class II (MHCII). At steady state ILC3s lack co-stimulatory molecule expression and appear to limit CD4^+^ T cell responses, however this interaction may be altered in inflammatory scenarios via upregulation of co-stimulatory molecules such as CD40, CD80, and CD86, which favor the promotion of T cell response. Furthermore, ILC3s act to modulate the survival of recirculating memory CD4^+^ T cells via interactions via OX40L and CD30L, although it is unknown whether this process also requires MHCII-dependent antigen presentation. In addition, ILC3 regulation of T follicular helper (TFH) cell responses has consequences for the priming of germinal center B cells and the induction of T-dependent IgA responses toward colon-dwelling commensal microbes. ILC3s can also modulate adaptive immune cells through the production of regulatory cytokines and growth factors. In line with this, ILC3 directly support B cell responses in the spleen through provision of critical growth factors such as BAFF/APRIL. Similarly, ILC3 also modulate the magnitude of the T cell response within the intestinal tract through the production of soluble mediators. For example, ILC3-derived IL-22 induces epithelial serum amyloid A (SAA) protein, which subsequently promotes local Th17 responses and acts to limit colonization with segmented filamentous bacteria (SFB) via the induction of antimicrobial peptides. In addition, ILC3 facilitate the establishment of a regulatory and tolerogenic environment in the gut by promoting regulatory T cell (Treg) responses. ILC3 crosstalk with tissue-resident myeloid cell populations establishes a feedback circuit whereby ILC3-derived GM-CSF promotes IL-10 and RA production by myeloid cells to promote Treg conversion. Conversely, Treg, myeloid cells and ILC3 may feedback on each other through a variety of soluble and cell-cell interactions suggesting a dynamic and malleable communication loop to ensure tolerance and tissue homeostasis. Finally, ILC3 subsets are a potent source of IL-2 in the small intestine that provides survival signals for Treg. Together these tissue-resident immune circuits place ILC3 at the center of a number of pathways through which they regulate adaptive immune responses to promote tissue health and homeostatic interactions with the microbiota.

As discussed above, ILC3s contribute to the formation of lymphoid structures and were found to be strategically positioned in clusters within lymph nodes where they have potential to interact with both T and B cells both directly and indirectly (127). Many of the same mechanisms employed by ILC3 to induce lymphoid organogenesis during early life are similarly employed in adult tissues to regulate the adaptive immune system. For example, ILC3s can support the production of IgA by B cells in the PP, in part through both soluble LTα_3_ and surface bound LTα1β2 interactions with DCs (128, 129). Similarly, in the spleen, production of LTα1β2, GM-CSF, and BAFF/APRIL production by ILC3s also acts to support B cell responses (Figure 3: *outputs*) (130).

In line with these findings, ILC3s have the capacity to crosstalk both directly and indirectly with the adaptive immune system through the production of multiple soluble factors. Following exposure to the commensal microbiota IL-22 produced by ILC3s acts to support homeostatic tissue Th17 responses through the induction of serum amyloid protein A (SAA) from epithelial cells (Figure 3: *outputs*) (131). Interestingly, ILC3 derived IL-22 can also prevent the activation of T cells in an AhR-dependent manner to limit immune activation or tissue damage (132). Conversely, T cells may also regulate the magnitude of ILC3-derived IL-22 production (26, 133), suggesting complex crosstalk between T cells and ILC3 in determining the level of IL-22 produced in the tissue.

As highlighted previously, sensing of the microbiota by myeloid cells is a critical regulator of ILC3 responses, and has consequences for adaptive immunity. IL-1β induction of GM-CSF production by ILC3s feeds back on tissue-resident MNP to trigger IL-10 and RA production by intestinal macrophages and DCs—resulting in the induction and maintenance of tissue regulatory T cells (Treg) and reinforcing immune tolerance (Figure 3: *inputs*/*outputs*) (134). Similarly, IL-1β produced by intestinal MNP further induces ILC3 to produce IL-2, a critical growth signal that helps to support peripherally induced Tregs in the small intestine and to maintain intestinal tolerance (Figure 3: *inputs*/*outputs*) (135). Conversely Treg interactions with MNP may limit IL-23 production to prevent ILC3-driven inflammation via a LAG3-dependent mechanism (Figure 3: *outputs*) (136), implicating a bidirectional axis involving ILC3, MNP, and Treg in determining the immune tone of the intestinal tract.

ILC3s are increasingly appreciated to also act as a direct orchestrator of tissue immune responses through their ability to act as antigen-presenting cells. ILC3 are also endowed with a broad array of accessory co-activating and co–inhibitory molecules that enable further modulation and tuning of adaptive immune cell function. Thus, when coupled with their strategic localization within lymphoid structures, ILC3 have the potential to potently regulate adaptive immune responses. At steady state, ILC3s in the mLNs and large intestine constitutively express MHC class II (MHCII) molecules at levels comparable with other professional antigen-presenting cells and can acquire, process and present antigens (Figure 3: *inputs*/*outputs*) (137). However, under homeostatic circumstances these interactions do not induce T cell proliferation, due in part to the absence of classical co-stimulatory molecules such as CD40, CD80, and CD86 on the cell surface (137). In contrast MHCII^+^ ILC3s were found to suppress effector CD4^+^ T cell responses toward the microbiota in the intestine (137–139). In line with a suppressive function for ILC3-associated antigen presentation, deletion of ILC3-intrinsic MHCII also disrupts crosstalk between ILC3 and adaptive immune cells at the interfollicular border of the mLN resulting in a spontaneous T follicular helper response that subsequently drives increased IgA responses against mucosal-dwelling commensals, and results in an altered intestinal metabolome (Figure 3: *outputs*) (120). While these findings suggest a suppressive and regulatory role for antigen-presenting ILC3 in the context of health, additional reports suggest that in contrast during immunization or infection tissue-specific inflammatory cues act to alter the nature—and consequences—of ILC3 antigen presentation. Indeed, activation of ILC3 by IL-1β resulted in antigen-presentation dependent *promotion* of T cell responses as a result of upregulated expression of classical co-stimulatory molecules (CD80/CD86) on ILC3 (140). In addition to antigen-presentation to CD4^+^ T cell subsets, ILC3 also express CD1d—conferring the ability to present lipid antigens to invariant (i) NKT cell populations, and promote their functionality (141).

Indeed, ILC3 have the capacity to modulate a broad variety of specialized adaptive immune responses through cell-cell interactions via additional non-classical co-stimulatory and co-inhibitory molecules. Seminal early studies in the field demonstrated a critical role for ILC3-associated CD30L and OX40L in the modulation of T cell memory through cognate interactions with CD30/OX40 (Figure 3: *inputs*/*outputs*) (126, 142, 143). Recent studies have expanded upon these observations to demonstrate a role for tissue-resident MNP-derived TL1A in regulating the expression of OX40L on ILC3, which was subsequently demonstrated to enable ILC3 to promote inflammatory effector T cell responses in the context of colitis (144). In addition ILC3 have been reported to express co-inhibitory and immune checkpoint molecules (e.g., PD1, PDL1) suggesting further immunoregulatory functions for these cells—although further investigation is required to determine the functional relevance of this receptor repertoire (145, 146). As investigation into this aspect of ILC3 function increases, the nature and breadth of interactions with both innate and adaptive immunity are likely to expand and present new intervention possibilities for the modulation of tissue immune responses.

## Conclusions and Future Perspectives

The maintenance of mucosal homeostasis is mediated through a complex interplay between the host and its environment, between immune and non-immune cells and by the balance of pathogenic and commensal microbes. Here we have highlighted the contributions of sensory circuits within the intestinal tract, which culminate in the activation and regulation of ILC3s. ILC3 display connectivity with an increasing number of physiological systems, many of which are likely to act simultaneously within the tissue in the context of health and disease—and ultimately to regulate the same range of ILC3-derived *outputs*. Thus, despite recent advances, one future challenge will be to understand how ILC3 integrate multiple concurrent signals from varying biological systems within a given tissue niche, and to determine how these cues are translated into cell fate decisions to determine the magnitude or quality of an ILC3 response. Many signaling pathways downstream of both cytokine and neuropeptide receptors converge upon core regulators of cell function—such as the mammalian target of rapamycin (mTOR) (147). Moreover, the appropriate licensing and modulation of anabolic cell metabolism pathways in order to generate new cellular biomass, effector proteins and facilitate proliferation is a central checkpoint of cellular function, critical to regulate immune cell function and controlled in part through mTOR activation (148). In line with this, a recent report demonstrated the induction of an mTOR complex 1-dependent programme of glycolytic metabolism as a central rate-limiting step in the production of ILC3-derived cytokines and proliferation (149). Engagement of glycolysis was also associated with the expression of the oxygen-sensing transcription factor HIF1α, suggesting other tissue-specific environmental factors may augment ILC3 responses via licensing of glycolysis and anabolic metabolism.

Ultimately, an increased knowledge of the network of *inputs* and *outputs—*and importantly the mechanisms through which these multiple sensory circuits are integrated and interpreted—will allow for new approaches to target this mucosal immune sentinel in the context of health and disease. Indeed, while ILC3 mediate many protective processes at homeostasis, dysregulated ILC3 responses have been implicated in a wide range of chronic inflammatory and metabolic diseases and have increasingly been suggested to play roles in cancer development and progression. Most notably, disruption of ILC3 responses is associated with the pathogenesis of inflammatory bowel disease (IBD) (1, 27, 40, 41, 62, 138, 150). Interestingly, lifestyles associated with disruption of sleep cycles and circadian rhythms (e.g., shift work, jet lag) have been suggested as potential triggers for IBD flares (151). Thus, while there have been recent major achievements in the understanding of how ILC3 sense signals from the CNS and ENS and perceive circadian cues (72, 74, 75, 95–97), the physiological impact of these systems on ILC3 function in the context of IBD could prove important in beginning to decode the multitude of factors that lead to disease onset and progression.

In conclusion, ILC3 are strategically positioned within mucosal sites where they act as a hub of multiple distinct, yet complementary, sensory circuits. Together, these circuits act to continually survey the intestinal tract for perturbations in microbial, dietary and external environmental cues and enable the rapid communication and translation of this information, resulting in protective effector responses that continually reinforce normal tissue function and health. Strategies aimed at exploiting these cues and sensory circuits to promote or restore homeostatic ILC3 function, while simultaneously suppressing the dysregulated signaling associated with maladapted immune function, may lead to novel therapeutic intervention strategies in a number of human diseases.

## Author Contributions

MH and RD conceived of and contributed to the writing of the manuscript. RD constructed the figures.

### Conflict of Interest

The authors declare that the research was conducted in the absence of any commercial or financial relationships that could be construed as a potential conflict of interest.
